# Hyper-spectral response and estimation model of soil degradation in Kenli County, the Yellow River Delta

**DOI:** 10.1371/journal.pone.0227594

**Published:** 2020-01-08

**Authors:** Chunyan Chang, Fen Lin, Xue Zhou, Gengxing Zhao

**Affiliations:** 1 National Engineering Laboratory for Efficient Utilization of Soil and Fertilizer Resources, College of Resources and Environment, Shandong Agricultural University, Tai’an, China; 2 Qingdao Hengyuande Real Estate Appraisal Limited Company, Qingdao, China; 3 Department of Agricultural and Biological Engineering, University of Florida, Gainesville, Florida, United States of America; Shandong University, CHINA

## Abstract

The ecological environment of the Yellow River Delta is fragile, and the soil degradation in the region is serious. Therefore it is important to discern the status of the soil degradation in a timely manner for soil conservation and utilization. The study area of this study was Kenli County in the Yellow River Delta of China. First, physical and chemical data of the soil were obtained by field investigations and soil sample analyses, and the hyper-spectra of air-dried soil samples were obtained via spectrometer. Then, the soil degradation index (SDI) was constructed by the key indicators of soil degradation, including pH, SSC, OM, AN, AP, AK, and soil texture. Next, according to a cluster analysis, soil degradation was divided into the following three grades: light degradation, moderate degradation, and heavy degradation. Moreover, the spectral characteristics of soil degradation were analyzed, and an estimation model of SDI was established by multiple stepwise regression. The results showed that the overall level of reflectance spectra increased with increased degree of soil degradation, that both derivative transformation and waveband reorganization could enhance the spectral information of soil degradation, and that the correlation between SDI and the spectral parameter of (R_λ2_+R_λ1_)/(R_λ2_-R_λ1_) was the highest among all the spectral parameters studied. On this basis, the optimum estimation model of SDI was established with the correlation coefficient of 0.811. This study fully embodies the potential of hyper-spectral technology in the study of soil degradation and provides a technical reference for the rapid extraction of information from soil degradation. Additionally, the study area is typical and representative, and thus can indirectly reflect the soil degradation situation of the whole Yellow River Delta.

## Introduction

Due to the large Chinese population and the increasingly smaller appropriation of per capita land resources, natural resources have been used unreasonably for a long time. In particular, unreasonable use of land resources has caused serious damage to regional ecological environments, consequently causing an increase of the severity of soil degradation [1, 2]. The Yellow River Delta, located on the west coast of the Bohai Sea, is an important land resource reserve in China. It has a fragile ecological environment and serious salinization degradation under the impact of the dynamic systems of rivers, land, ocean, and other environmental factors [3–5]. Soil degradation restricts sustainable development of the economy and society; consequently, it is a very urgent task to address the soil degradation status in a timely manner and utilize and protect soil resources in the area rationally.

Spectroscopy technology can obtain surface information quickly and has many obvious advantages in speed and cost. Many scholars around the world have used satellite-based spectra to study soil degradation. For example, Cavalli et al. used Advanced Very High Resolution Radiometer (AVHRR) data to evaluate soil erosion in the middle-eastern region of the state of São Paulo, Brazil [6], and Wang et al. studied the problem of soil and water loss using Landsat TM in the Loess Plateau, China [7]. AVHRR images from the NOAA meteorological satellite, which are updated quickly and cover a large area, can often be used to monitor large-scale vegetation growth through the calculation of a vegetation index and thus can be used to infer the degree of soil degradation indirectly [8–10]. Compared with AVHRR, which has a scan width of 2800km, the coverage width of the Landsat satellite, with a scan width of 185 km, is smaller, the spatial resolution is significantly improved, and data acquisition is convenient. TM and ETM+ images are used widely in the study of middle-scale soil erosion, salinization and desertification [11–13]. SPOT satellite data have advantages in spatial resolution and are often used in soil degradation mapping, even though SPOT has fewer bands. However, the spectral width of the multi-spectral data is generally greater than 100 nm, the bands are not continuous in the spectrum, the number of bands is small, and it is difficult to cover the range of the entire visible and infrared spectrum. In addition, the different degrees of soil degradation are difficult to quantify accurately. However, with the expansion of soil research, high-resolution spectral data have gradually revealed advantages. The spectra, with their narrow and high-resolution bands, have great potential, especially in the quantitative study of soil properties [14, 15].

The hyperspectral curves of soils, which are acquired within the visible to near-infrared spectral range, are almost consecutive, and their band widths are less than 10 nm. Thus, inversion for higher accuracy of land details is possible. The electromagnetic radiation energies of soil properties including soil organic matter, texture, carbonate content, and iron oxide content, are stronger, and the characteristics of spectroscopic diagnosis are obvious. Through physical-chemical data analysis, after identifying sensitive bands or characteristics of spectroscopic diagnosis, quantitative inversion can be carried out directly. Therefore, there are many studies on soil degradation related to organic matter deficiency, salinization and desertification [16–18]. To realize precision agriculture, it is of great significance to monitor the three elements of nitrogen, phosphorus, and potassium quickly, as these elements are the three essential nutrients of crops. Nevertheless, research has shown that it is difficult to estimate these three elements directly by spectral feature analysis. DeTar et al. detected soil properties of bare fields with airborne hyperspectral data, which was located on the western side of the San Joaquin Valley of California and the soil was silty clay loam (slight to strong alkali), the results showed that phosphorus acquisition was not satisfactory but that potassium acquisition was better. Confalonieri et al. used near-infrared spectra to determine the properties of agricultural soils, which lied in the Po valley near Lodi (Northern Italy) on sandy loam soil, the results showed that nitrogen content could be determined accurately, but the determinations of potassium and phosphorus contents were less successful. The validity of the method based on spectroscopic estimation of soil nitrogen, phosphorus and potassium remains controversial [19, 20], which is why there are few studies on soil degradation caused by soil nutrient deficiency.

Soil degradation is a dynamic and complex process. Its causes are often complex, but the main cause of soil degradation is the joint action of natural and social factors, such as erosion, desertification, salinization, acidification, etc. It is dangerous for humans to exploit and utilize agricultural resources blindly, such as by deforestation, overgrazing, unreasonable farming, etc., and it is difficult to reflect the real situation of soil degradation when considering only one reason for degradation. However, the soil spectrum is a comprehensive reflection of all soil properties, so study of the spectral characteristics of soil degradation under the action of multiple factors is more scientific [21]. Among previous studies, there have been many studies on single types of soil degradation, such as soil pollution, erosion, salinization and organic matter deficiency. Mathieu et al. studied soil erosion with SPOT-HRV images in central Chile [22]. Cannane et al. studied polluted soils by FT-IR spectral data in the Puducherry State of South India [23]. Guo et al. studied soil salinization with Landsat5 TM and Landsat8 OLI images in the Yellow River Delta [24]. Mirzaee et al. studied soil organic matter using Landsat7 ETM+ in the Selin plain of Iran [25]. However, there have been relatively few comprehensive studies on multiple indicators of soil degradation.

Most of the studies of hyper-spectral soil degradation in the Yellow River Deltafocus on soil salinization, and which are partial to the study method of information extraction. Moreover, there is little research on soil nutrient impoverishment, and study of the hyper-spectral response of soil degradation remains insufficient [26–29]. On one hand, the research methods are immature, and the results are still objectionable. On the other hand, the spectral data of field soil samples, which are still limited in their application, are ineffective and must be processed indoors. In addition, to present, many theoretical problems and process mechanisms of soil degradation remain unclear, and there are no recognized or unified soil degradation indicators. Therefore, it is of great academic value to carry out spectral estimation of soil degradation and the establishment of a degradation indicators system that can provide a scientific basis for the protection of the soil environment and the rational planning and utilization of land resources in the Yellow River Delta region.

In this study, a soil degradation index (SDI) was constructed according to several key indicators of soil degradation. The spectral characteristics of soil degradation under the action of multiple factors were analyzed through the hyperspectral data collected by ASD FieldSpec4, and estimation models of SDI were constructed. The purpose of the study was to investigate the hyper-spectral response and quantitative estimation of soil degradation in typical areas of the Yellow River Delta, which could provide a basis for the rational formulation of agricultural policies in the Yellow River Delta and could have important practical significance for improving soil quality in the delta.

## Materials and methods

### Study area

This study was performed in Kenli County (37°24′-38°10′ N, 118°15′-119°19′ E) in the Yellow River Delta of China, which belongs to Dongying City, Shandong Province. As the major source of freshwater and groundwater recharge, the Yellow River flows from the southwest of Kenli County to the northeast into the Bohai Sea. There are high water tables and high degrees of groundwater mineralization. The regional groundwater depths of 44.1% of the county area are from 1–2 m, and those of 7% the county area are less 1 m. The average degree of groundwater mineralization is 24.6 g/l, and the highest value is up to 167.5 g/l [30]. The main landforms include coastal lowland, gentle slope, and low-lying land, among others. The main soil type is saline soil derived from alluvial deposits of the Yellow River, which has a light loam soil texture and nutrient deficiencies. Soil degradation in the region manifests mainly as salinization, alkalization, and diminishing fertility, which cause negative effects to the local agricultural production [31–33]. The local crops include mainly winter wheat (Triticum aestivuml), corn (Zea mays), paddy (Oryza sativa), and cotton (Gossypium), and most of the natural vegetation consists of salt-tolerant herbaceous plants and shrubs, such as reed (Phragmites australis), cogongrass (Imperata cylindrica), seepweed (Suaeda glauca), and salt cedar (Tamarix chinensis).

### Soil sampling and laboratory analyses

**Fig 1:**
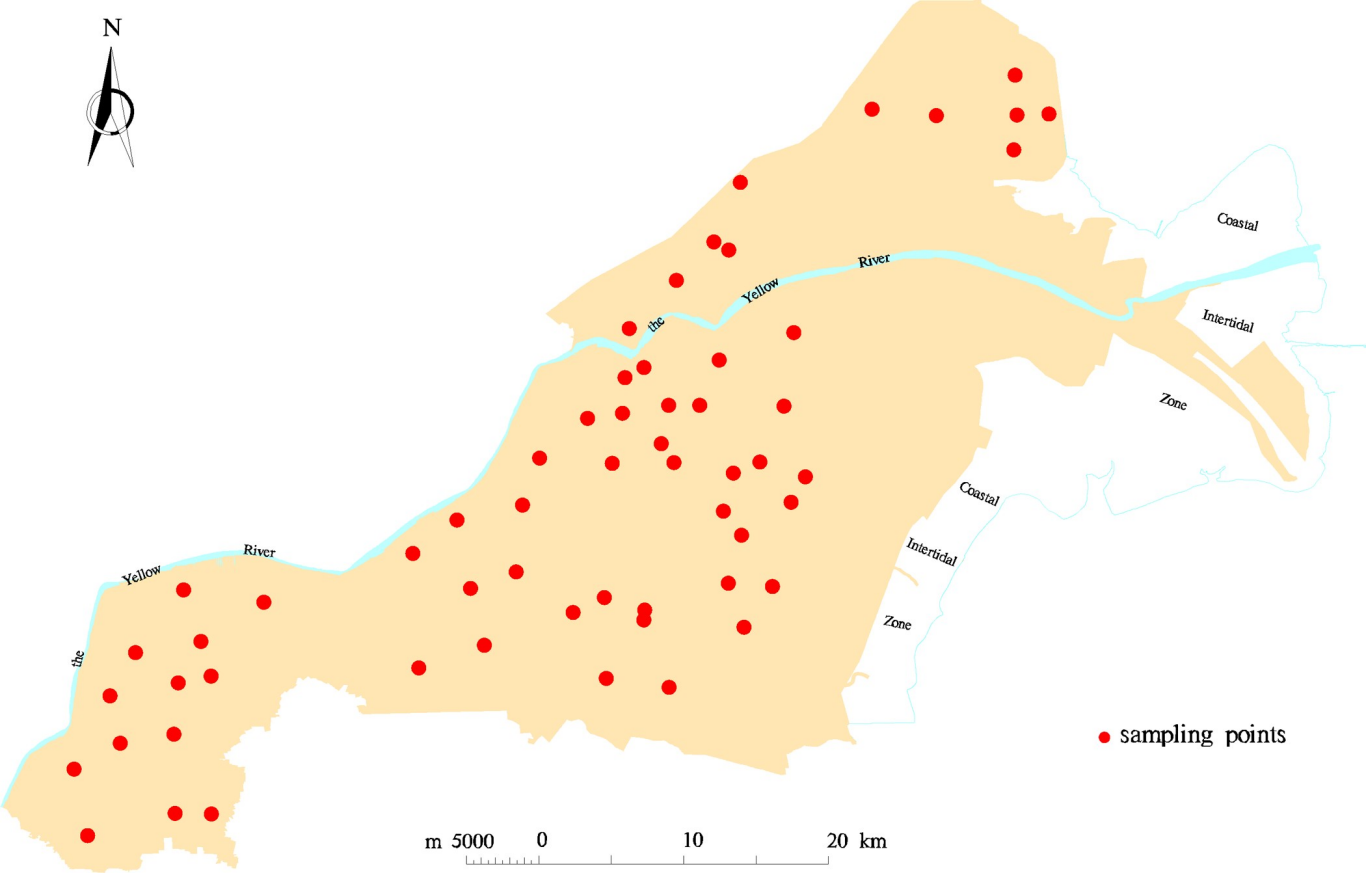
Distribution map of the sampling points.

Soil samples were collected in the study area from April 23 to 25, 2015. Fifty-nine long-term, fixed-point observation points were distributed evenly in Kenli County, except for the coastal intertidal zone, as shown in Fig 1, and we added a number of samples at some observation points with more types of vegetation on the surface. Eventually, we obtained 71 soil samples from the 59 observation points. Each soil sample had a mass of approximately 1 kg. The samples were collected from the 0 to 20 cm interval of surface soil and then placed into a sealed bag. The specific information of the soil samples, such as sample number, geographical coordinates, land use types, vegetation cover types, vegetation growth, soil types, and texture, was recorded.

Mixed soil samples from multiple points were brought back to the laboratory, where they were dried, ground and sieved through 2 mm soil sieves. Laboratory tests were performed to obtain the soil chemical properties, which included pH, soil salt content (SSC), organic matter (OM), available nitrogen (AN), available phosphorus (AP) and available potassium (AK). PH and SSC were measured using a WTW inoLab® Multi 3420 Set B multiparameter measuring instrument [34]. OM was determined by the potassium dichromate method [35]. AN was determined by the method of alkaline hydrolysis diffusion [36]. AP was estimated by sodium bicarbonate extraction method [37]. AK was extracted using 1 mol/L ammonium acetate (pH = 7.0) and determined by the ammonium acetate method [38].

### Soil degradation index

#### Indicators for soil degradation evaluation

**Fig 2:**
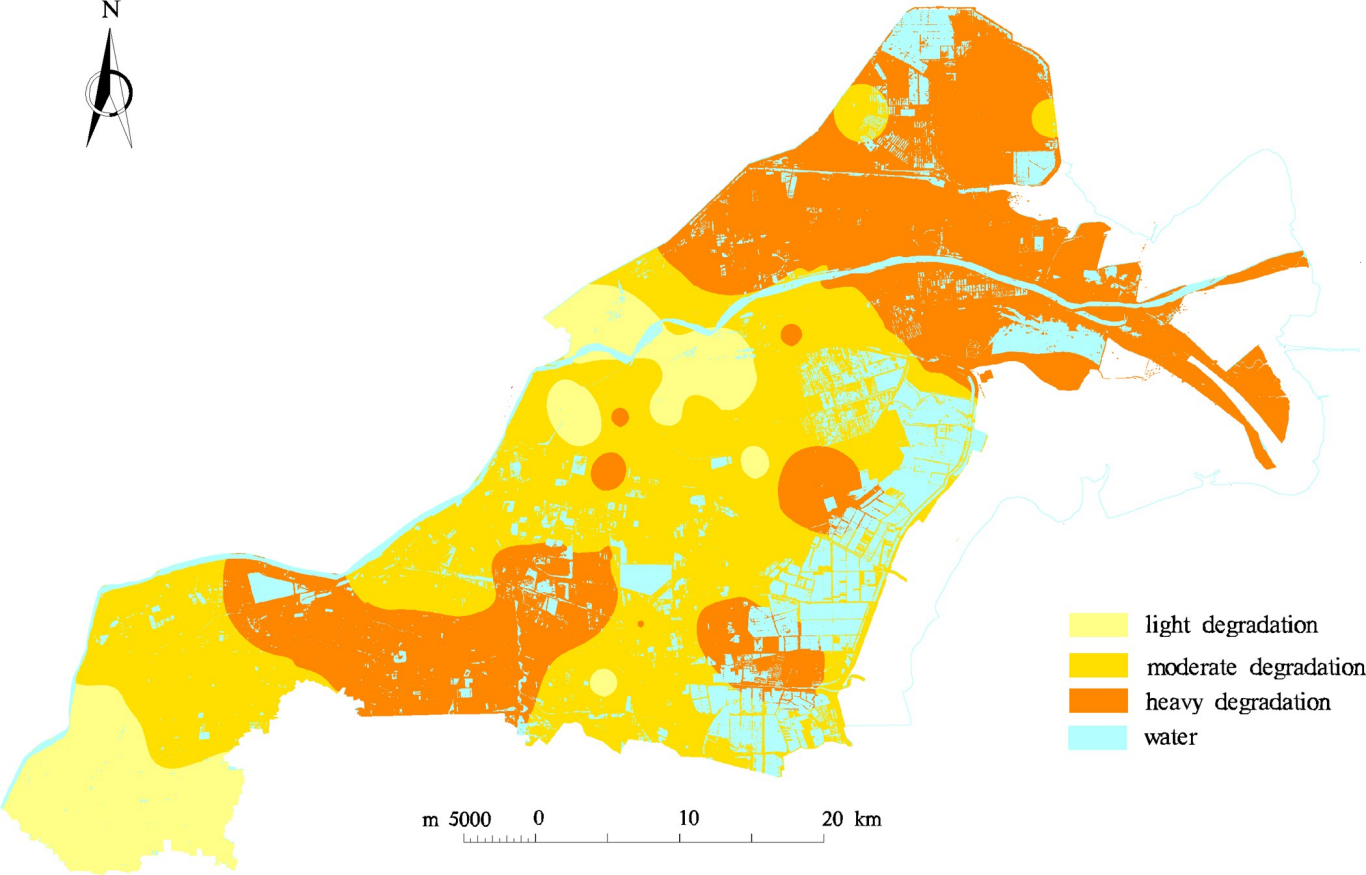
Interpolation map of soil degradation.

Many factors affect soil degradation, and there is no uniform standard at present to evaluate the degree of soil degradation. Soil degradation in Kenli County is mainly manifest as salinization, alkalization, and declining fertility, so pH, SSC, OM, AN, AP, AK, and soil texture were used as evaluation indicators of soil degradation in this study. The value of pH was used to illustrate the soil alkalization, SSC was used to illustrate the soil salinization, OM and available nutrients were used to illustrate the level of soil fertility, and soil texture was used to illustrate the soil physical properties. According to the experiences of experts, soil texture has been transformed from a qualitative description to a quantitative value, and the quantitative values of medium loam, light loam, heavy loam, clay loam, and sandy loam are 100, 95, 90, 85 and 75, respectively [39]. Table 1 presents the statistical characteristics of the evaluation indicators. The SDI values of the sample points were spatially interpolated, graded, and colored according to SDI grading standards, and the interpolation map of the soil degradation based on the field data was generated using the inverse distance weighted interpolation method in ArcGIS, as shown in Fig 2.

**Table 1:** Statistical characteristics of evaluation indicators.

	Range	Average	Standard deviation
pH	7.13–8.34	7.78	0.29
SSC (g/kg)	0.11–26.11	5.32	5.76
OM (g/kg)	3.14–30.87	12.78	6.12
AN (mg/kg)	18.13–90	49.09	18.51
AP (mg/kg)	0.31–59.6	17.69	15.16
AK (mg/kg)	76.25–481	212.12	100.41
Soil texture	75–100	91.59	8.14

Note: SSC: soil salt content; OM: organic matter; AN: available nitrogen; AP: available phosphorous; AK: available potassium.

#### Data standardization

When the value of soil pH is approximately seven, the soil salt content is relatively low, and the contents of OM and available nutrients are relatively high, the quantitative value of soil texture is relatively high. In this situation, soil degradation is relatively mild and soil quality is better. The trends and dimensions of the evaluation indicators differ, so each indicator needs to be treated by data normalization to achieve the same dimensionless trend.

By combining the classification standards of the Second National Survey of Soil Nutrients in China [40], the Chinese Classification Standards of Soil Salinization[41], and the statistical characteristics of the evaluation indicators, the reference soil was set as follows: 6.5 ≤ pH ≤ 7.5, SSC ≤ 1.0 g/kg, OM ≥ 20 g/kg, AN ≥ 90 mg/kg, AP ≥ 40 mg/kg, AK ≥ 200 mg/kg, and soil texture = 100. Then, the values of the evaluation indicators were standardized by using the distance standardization method. Thus, if the value of an evaluation indicator was farther from the standard soil, the standardized value was greater.

#### Soil degradation index

The entropy weight method has been used to determine the weight of evaluation indicators in many studies of soil quality due to its characteristic of being able to distinguish each evaluation indicator clearly. It can be used easily and obtains objective results [42, 43]. The weights of the evaluation indicators were determined by the entropy weight method in this study, and the SDI was calculated using the following equation:

$$\text{SDI}=\sum F_{i}\times W_{i}$$ (1)

where *F* is the standardized value of each evaluation indicator *i*, and *W* is the weighting factor of each evaluation indicator *i*. As SDI increases, the seriousness of soil degradation increases.

### Spectral measurements and processing

#### Spectral measurements and preprocessing

The hyperspectra of the soil samples were acquired by a FieldSpec4 spectrometer (Analytical Spectral Devices, Inc., Boulder, CO, USA) with a probe-viewing angle of 25°. The wavelength range of this spectrometer is 350–2500 nm, in which the wavelength range of 350–1000 nm has a sampling interval of 1.4 nm and a spectral resolution of 3 nm and the wavelength range of 1000–2500 nm has a sampling interval of 2 nm and spectral resolution of 8 nm. The spectrometer has a resampling interval of 1 nm and a total of 2151 output wavelengths.

The hyper-spectra were collected from air-dried soil samples under the conditions of cloudless, stable natural light. The measurement was conducted from 10:00 to 14:00 LST, and the spectrometer performed a white reflectance standard every 10 min during measurement. Each air-dried soil sample was placed in a plastic sample container (2 cm in depth; 10 cm in diameter), the soil surface was flattened slightly, and then the soil was placed 15 cm below the probe. To reduce the influence of the direction of the probe on the spectra, each soil sample was collected 4 times and measured once every 90°. Each measurement had five spectral curves, so each soil sample resulted in 20 original spectral curves. Then, any abnormal curve from the 20 original reflectance spectra was removed, the arithmetic average of the normal curves was calculated, and the nine-point moving average method was used to reduce the noise of the average curves. Thus, the reflectance spectra of the soil sample was eventually obtained.

#### Screening sensitive wavebands and constructing spectral parameters

The first derivative of the spectra can remove the effect of background noise and rapidly locate the inflection point of the reflectance spectra, so the spectral information can be enhanced by a first derivative transformation [44]. Therefore, in this study, the reflectance spectra were converted to the first derivative. The reflectance spectra and first derivatives of the spectra were used to analyze the correlation with SDI, and the peaks and troughs of the correlation coefficient curves were regarded as the positions of sensitive wavebands.

Specific spectral parameters can eliminate the influence of background noise, enlarge the difference between wavebands, and improve the precision of the inversion model. According to the spectral characteristics of the different levels of soil degradation, several forms of waveband recombinations were designed by binary operations such as addition, subtraction, multiplication and division. Then, spectral parameters were produced by substituting sensitive wavebands into those waveband recombinations. The sensitive bands, which have larger correlations with SDI, will be used to build models.

### Model development for the estimation

In this study, the reflectance spectra, first derivatives of the spectra, and spectral parameters were independent variables, and SDI was the dependent variable. The estimation models of SDI were established by using a stepwise multiple linear regression method based on various independent variables in IBM SPSS Statistics 19.0 software (IBM, Inc., Armonk, NY, USA).

Approximately 75% (total of 53) of the soil samples was randomly selected from each degradation level and used to establish the model. The remaining soil samples (total of 18) were used to evaluate the model accuracy. The availability of the model was tested by the significance test (sig.) value of the independent variable, and the independent variable made a significant contribution to the dependent variable when the sig. value was less than 0.05. Only when the sig. values of the independent variables in the model were less than 0.05 was the model qualified. Each stepwise multiple linear regression process produced many fitting equations, so the equation with the largest adjustment coefficient (adjusted R^2^) was chosen as the final result. The goodness of fit of the models was measured by the coefficient of determination (R^2^), the root mean square error (RMSE), and the ratio of performance to deviation (RPD). Higher values of R^2^ and RPD and lower value of RMSE indicate better model fit. When RPD was higher than 2, the model had high reliability and good performance; when RPD was higher than 1.4 but lower than 2, the prediction of the model was good but the model required improvement; when RPD was lower than 1.4, the model was not reliable and was unable to predict the samples[45,46].

## Results and discussion

### Analysis of soil degradation evaluation

The entropy weight method was adopted to determine the weights of the index. Supposing that there are k evaluation indexes and that each index has n values representing n soil samples, Y_ij_ represents the ith value of the jth evaluation index, R = (Y_ij_)_n×k_ (i = 1,2,…n; j = 1,2,…k) is the normalized matrix after dimensionless treatment, P_ij_ represents the proportion of Y_ij_ to the sum of all the index values, E_j_ represents the information entropy of the jth index, and W_j_ represents the weight of the index. The weights of the index are calculated as follows.

1. Data standardization is carried out for each index, and a standardization matrix is obtained.
  $$\text{R}={\left(Y_{ij}\right)}_{n\times k}$$ (2)
2. The information entropy of indicators is determined by formula (3).
  $$E_{j}=-\frac{\Sigma_{i=1}^{n}P_{ij}LnP_{ij}}{Ln\left(n\right)}$$ (3)
  wherein
  $$P_{ij}=\frac{Y_{ij}}{\Sigma_{i=1}^{n}Y_{ij}}$$
  and if ${\text{P}}_{\text{ij}}=0$, $\lim_{P_{ij}\to 0}P_{ij}LnP_{ij}=0$.
3. The weight of the jth index is determined as follows.
  $$W_{j}=\frac{1-E_{j}}{K-\Sigma_{j=1}^{k}E_{j}}\left(\text{j}=1,2,\ldots \text{k}\right),\Sigma_{j=1}^{k}W_{j}=1.$$

The entropy weighting method obtained the weight value of each index: pH = 0.1446, SSC = 0.1874, OM = 0.1412, AN = 0.1407, AP = 0.1399, AK = 0.1370 and soil texture = 0.1092, as shown in Table 2. The weights of SSC was relatively large, followed by PH, which iss consistent with the fact that the soil in Kenli County is affected by salinity and alkalinity.

**Table 2:** Weights of evaluation indicators.

pH	SSC	OM	AN	AP	AK	Soil texture
0.1446	0.1874	0.1412	0.1407	0.1399	0.1370	0.1092

Note: SSC: soil salt content; OM: organic matter; AN: available nitrogen; AP: available phosphorous; AK: available potassium.

The SDI of the soil samples ranged from 0.2366 to 0.6590. According to the case of local soil degradation and the result of a cluster analysis and based on previous studies [47, 48], soil degradation was divided into the following three grades: SDI < 0.40, light degradation; 0.40 < SDI < 0.54, moderate degradation; and 0.54 < SDI, heavy degradation. As a result, there were 28 (approximately 39% of the total) soil samples with light degradation, 35 (approximately 50% of the total) with moderate degradation, and 8 (approximately 11% of the total) with heavy degradation. Table 3 shows the land use types of the sample points with different soil degradation grades.

**Table 3:** Land use types of the sample points with different soil degradation grades.

		Low degradation	Moderate degradation	High degradation
Vegetable field		2		
Wheat field		14	7	
Cotton field		8	14	
Paddy		1	5	
Terek bostan		1	2	
Wasteland	Cogongrass	2	2	
	Reed		3	
	Seepweed		2	2
	Bare land			6

Obviously, soil samples that showed light degradation came mostly from wheat and cotton fields, and soil samples that showed moderate degradation came mostly from cotton fields, wheat fields and paddies. Soil samples that showed heavy degradation were collected from wasteland, which included only the bare land and seepweed land cover types. The field investigation showed that most wheat fields were lightly degraded. The above analysis illustrates that salinization is the main factor of soil degradation in Kenli County and that the vegetation cover types of the soil degradation grades from severe to mild were as follows: seepweed, reed, cogongrass, rice and cotton, and wheat [49].

### Spectral characteristics of soil degradation

**Fig 3:**
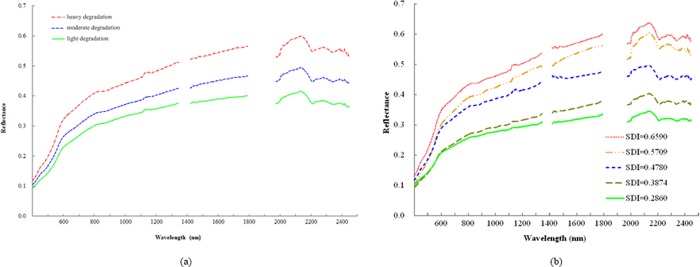
Average reflectance spectra of the different soil degradation grades (a) and reflectance spectra of soils with an SDI interval of approximately 0.1 (b).

After the spectral measurements and processing, 1835 wavebands were retained, including 354–1345, 1421–1791, and 1975–2446 nm. Fig 3A shows the average curves of the reflectance spectra in different soil degradation grades. The average SDI values of heavy degradation, moderate degradation, and light degradation were 0.5738, 0.4562, and 0.3528, respectively. The average difference of SDI of adjacent degradation grades was approximately 0.1. Fig 3B lists the reflectance spectra of the soil SDI interval of approximately 0.1 for further study of the spectral characteristics of the degraded soils.

From the aspect of waveforms, all waveforms were similar and had clear common features, as shown in Fig 3. In the range of 354–600 nm, the curves were steep, and the spectral reflectance increased rapidly with increase in wavelength. In the range of 601–1791 nm, the spectral curves rose steadily, and spectral reflectance increased with increase of wavelength. When the wavelength was greater than 1791 nm, spectral curves clearly fluctuated, and the positions of peaks and troughs were relatively stable. Spectral reflectance decreased as wavelength increased in several small ranges (2140–2210, 2275–2340, and 2385–2446 nm).

**Fig 4:**
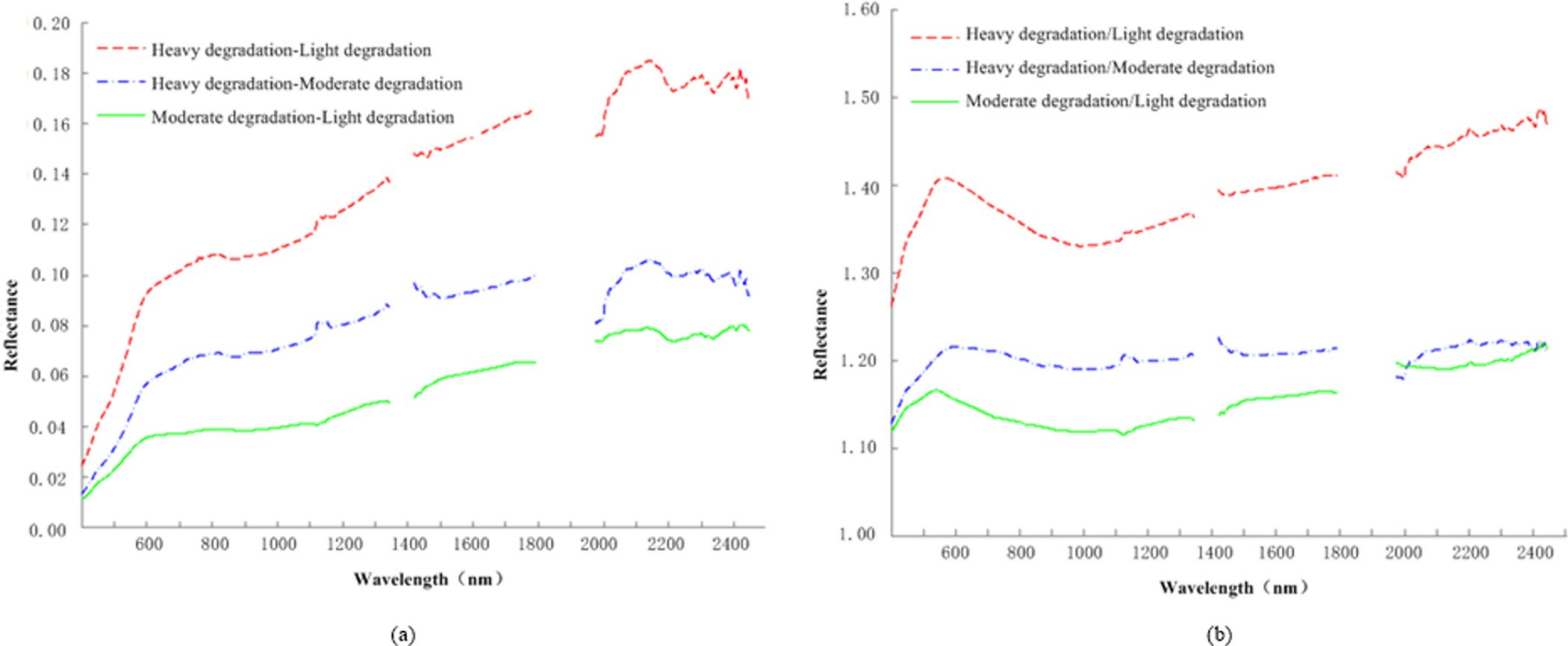
**Difference curves (a) and ratio curves (b).** Difference curves (a) show the difference of the average curves, which indicated the absolute quantity of reflectance between every two soil degradation grades. Ratio curves (b) show the ratio of average curves, which indicated the relative quantity of reflectance between two soil degradation grades.

From the aspect of the differences in reflectance, the soil of a superior degradation grade had higher spectral reflectance than the soil of an inferior degradation grade. The soil reflectance spectra in different degradation grades were not very different when the wavelength was less than 600 nm, but as the wavelength increased, the difference began to increase. To explore the relationship between SDI and soil reflectance further, the average curves of the reflectance spectra in different soil degradation grades were operated on using subtraction and division. Fig 4A shows the difference of the average curves, which indicates the absolute quantity of reflectance between every two soil degradation grades. Fig 4B shows the ratio of the average curves, which indicates the relative quantity of reflectance between two soil degradation grades.

The shapes among the difference curves and reflectance spectra were similar. At the position of maximum spectral reflectance (2140 nm), the difference of spectral reflectance was also largest. Therefore, the spectral information of soil degradation is more prominent in wavebands of larger spectral reflectance. The SDI values of heavy degradation and moderate degradation differed by 0.1023, and the SDI values of moderate degradation and light degradation differed by 0.0755. The differences of SDI between ‘heavy degradation and moderate degradation’ and ‘moderate degradation and light degradation’ (Fig 4A) were close, but the difference between the spectral reflectances was larger. This result means that the spectral characteristics of soil degradation are more obvious when the soil degradation is more serious.

In the 370–450 nm range, the ratio curves were steepest and the ratio changed most rapidly. Peaks, which were more affected by soil degradation than other wavebands, appeared within 500–650 nm. Peaks of ratio curves of ‘heavy degradation/light degradation’, ‘heavy degradation/moderate degradation’ and ‘moderate degradation/light degradation’ (Fig 4B) were located near 563, 595–600, and 540 nm, respectively. When the wavelength was less than 1975 nm, ‘heavy degradation/moderate degradation’ was higher than ‘moderate degradation/light degradation’, showing that there was a more clear impact on the reflectance spectra when the soil degradation was more serious, which is the same as in the previous analysis. When the wavelength was greater than 1975 nm, the ratio curves of ‘heavy degradation/moderate degradation’ and ‘moderate degradation/light degradation’ were close to each other, so the rate of change of spectral reflectance was similar between adjacent degradation grades; thus, there was a stable linear relationship between SDI and reflectance spectra in this range.

Clearly, the intervals of 370–450 nm, 500–650 nm and the wavebands of large spectral reflectance in the near-infrared range, especially the near long wave infrared range of wavelengths greater than 1975 nm, showed a good response to soil degradation, but these wavebands have a large number and are over a wide range; thus, further screening of sensitive wavebands of soil degradation is needed.

### Sensitive wavebands

**Fig 5:**
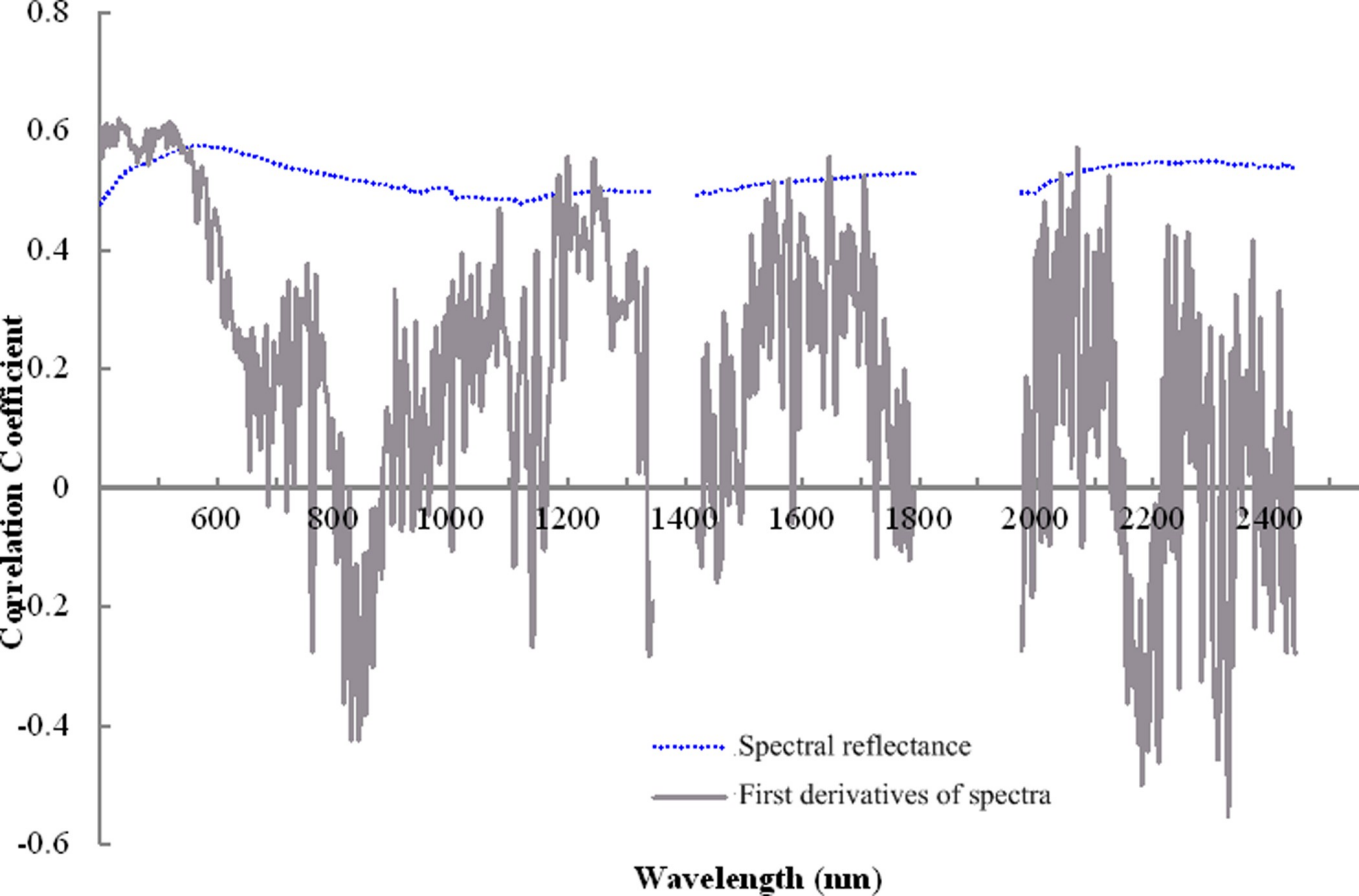
Correlation curves. The figure shows the correlation curve between reflectance spectra and SDI, and the correlation between the first derivative of spectra and SDI.

Fig 5 shows the correlation curve between reflectance spectra and SDI and the correlation between the first derivative of spectra and SDI. The correlation coefficient between the reflectance spectra and SDI was positive, and the value ranged from 0.45 to 0.60. The correlation coefficient was larger in the range of 560–600 nm, with a maximum value of 0.585 at 582 nm. This curve was relatively smooth, and it only had a slight sag at 934–936 nm. The correlation coefficient between the first derivative of spectra and SDI ranged from -0.56 to 0.65, and the correlation coefficient was larger in the ranges of 356–450 and 520–523 nm; it had a maximum value of 0.638 at 386 nm. This curve fluctuated strongly, and the wavebands of remarkable peaks and troughs occurred at 356, 371, 375, 563, 581, 583, 599, 656, 761, 773, 830, 955, 958, 1003, 1083, 1199, 1244, 1322, 1459, 1461, 1585, 1642, 1721, 1726, 1753, 2011, 2042, 1995–1997 2070, 2126, 2134, 2140, 2256, 2290, 2295, 2297, 2298, 2332, 2320, 2338, 2352, 2364, 2377, 2398, 2403, 2427, and 2441 nm.

To maximize the sensitive information of soil degradation, most of the relatively sensitive wavebands were preserved in the study, thus, the sensitive wavebands of soil degradation were determined to be 356, 371, 375, 386, 563, 581, 582, 583, 599, 656, 761, 773, 830, 934–936, 955, 958, 1003, 1083, 1199, 1244, 1322, 1459, 1461, 1585, 1642, 1721, 1726 1753, 1995–1997, 2011, 2042, 2070, 2126, 2134, 2140, 2256, 2290, 2295, 2297, 2298, 2332, 2320, 2338, 2352, 2364, 2377, 2398, 2403, 2427, and 2441 nm (a total of 54 wavebands).

### Spectral parameters

Based on the above analyses of the spectral characteristics of soil degradation, it can be seen that it is easier to highlight the spectral information of soil degradation using the bands that have high spectral reflectance and greater differences among them, which are conducive to the enhancement and extraction of soil degradation information. Therefore, the following five types of waveband recombination were designed: R_λ2_-R_λ1_, R_λ2_+R_λ1_, R_λ2_/R_λ1_, (R_λ2_-R_λ1_)/(R_λ2_+R_λ1_), and (R_λ2_+R_λ1_)/(R_λ2_-R_λ1_). Among these, λ1 and λ2 (λ2> λ1) are sensitive wavebands, and R is the spectral reflectance of sensitive wavebands. Spectral parameters were produced by substituting the 54 sensitive wavebands into these waveband recombinations. Finally, 1431 spectral parameters were obtained in each waveband recombination.

The correlation between spectral parameters and SDI was analyzed, and the first 100 spectral parameters with larger correlation coefficients in each waveband recombination were used to build the estimation model. As shown in Table 4, the spectral parameters of (R_λ2_+R_λ1_)/(R_λ2_-R_λ1_) correlated best with SDI.

**Table 4:** First 100 spectral parameters with a large correlation with SDI.

Type of spectral parameter	Maximum	Minimum	Average
R_λ2_-R_λ1_	0.586	0.524	0.542
R_λ2_+R_λ1_	0.588	0.584	0.586
R_λ2_/R_λ1_	0.626	0.566	0.579
(R_λ2_-R_λ1_)/(R_λ2_+R_λ1_)	0.578	0.515	0.539
(R_λ2_+R_λ1_)/(R_λ2_-R_λ1_)	0.664	0.613	0.622

The maximum correlation coefficients of SDI with reflectance spectra, the first derivative of spectra, and the spectral parameters were 0.585, 0.638, and 0.664, respectively. Therefore, the first derivative transformation and waveband reorganization was able to enhance soil information to some extent and improve the correlation with SDI.

### Estimation models for SDI

With the reflectance spectra, the first derivative of the spectra, and the spectral parameters as the independent variables, estimation models of SDI were established by stepwise multiple linear regression. The fitting equation with the largest adjusted R^2^ was chosen as the estimation model in each analysis of the stepwise multiple linear regression. The models obtained are shown in Table 5. The sig. values of all independent variables in Table 4 were less than 0.005, which passed the significance test, and thus these models were considered qualified models.

**Table 5:** Estimation models of SDI.

Independent variable		No.	Number of independent variables	Model accuracy		Fitting accuracy	
				R^2^	Adjusted R^2^	RMSE	RPD
Reflectance spectra	R	I	3	0.552	0.531	0.058	1.578
First derivative of spectra	R’	II	7	0.785	0.760	0.041	2.222
Spectral parameters	R_λ2_/R_λ1_	III	2	0.509	0.494	0.059	1.547
	R_λ2_-R_λ1_	IV	3	0.557	0.536	0.057	1.608
	R_λ2_+R_λ1_	V	3	0.563	0.543	0.056	1.626
	(R_λ2_-R_λ1_)/(R_λ2_+R_λ1_)	VI	3	0.515	0.492	0.062	1.478
	(R_λ2_+R_λ1_)/(R_λ2_-R_λ1_)	VII	7	0.811	0.786	0.039	2.369

The models of I, III, IV, V and VI had smaller R^2^ and larger RMSE than the other models, and RPD was only slightly larger than 1.4; thus, these models can roughly estimate SDI but are not sufficiently practical. The evaluation indices of models II and VII were better. These had more rational R^2^ and RMSE, and the RPD was greater than 2; thus, these models demonstrated good prediction ability for SDI. After a comprehensive comparison of R^2^, RMSE, and RPD, the modeling effects in the order from priority to inferiority were VII > II > V > IV > I > III > VI.

From the aspect of independent variables, modeling using the first derivative of spectra was better than modeling using reflectance spectra. The models that used different types of waveband reconstruction showed great differences in goodness of fit. Modeling using (R_λ2_+R_λ1_)/(R_λ2_-R_λ1_) was better than that using reflectance spectra or using the first derivative of spectra, modeling using R_λ2_+R_λ1_ or R_λ2_-R_λ1_ was better than that using reflectance spectra but worse than using the first derivative of spectra, and the goodness of fit of modeling using R_λ2_/R_λ1_ or (R_λ2_-R_λ1_)/(R_λ2_+R_λ1_) was worse than the others. From the aspect of the number of independent variables, model III had only two independent variables, models II and VII had seven independent variables, and the others had three independent variables. Therefore, the spectral information of soil degradation can clearly be enhanced by first derivative transformation and the operation of the sum of two wavebands divided by their difference. The addition operation and subtraction operation between two wavebands can enhance the information of soil degradation to some extent, but it was not significant enough. The division operation and the operation of the difference of two wavebands divided by their sum were not conducive to expressing the spectral information of soil degradation.

From the perspective of the wavebands involved, all estimation models in Table 5 involve 38 wavebands, including 8 visible bands, 8 near-infrared short wavebands, and 24 near-infrared long wavebands. Independent variables from models II and VII were composed mainly of near-infrared long wavebands. Thus, the sensitive wavebands of soil degradation were concentrated in the near-infrared long wave region.

For the estimation model of SDI constructed by (R_λ2_+R_λ1_)/(R_λ2_-R_λ1_), the R^2^ was 0.811, the RMSE was 0.039, and the RPD was 2.369. They were the optimal values in all models, so the optimum estimation model of SDI in this study was demonstrated to be Y = 0.684–0.306(R_581_+R_356_)/(R_581_-R_356_)+0.065(R_934_+R_599_)/(R_934_-R_599_)+7.383×10^−6^(R_2295_+R_1726_)/(R_2295_-R_1726_)-5.627×10^−5^(R_2403_+R_2011_)/(R_2403_-R_2011_)-5.661×10^−5^(R_2352_+R_1459_)/(R_2352_-R_1459_)-3.905×10^−6^(R_2290_+R_1996_)/(R_2290_-R_1996_)+2.561×10^−5^(R_2403_+R_1721_)/(R_2403_-R_1721_). The model consists of seven independent variables, which are composed of 14 wavebands, among which 10 wavebands are near-infrared long wavebands. The spectral parameters of (R_λ2_+R_λ1_)/(R_λ2_-R_λ1_) enlarge the difference between two wavebands using the division operation of their sum divided by their difference; thus, the information of soil degradation is enhanced and the established estimation model of SDI is efficient and stable.

The results show that the hyperspectral data can be applied to the estimation of soil degradation, but this research is not extensive and the results are not universal. Additionally, the applicability of the spectral data is restricted to the coastal areas of the Yellow River Delta. Compared with previous studies, which focused mainly on one type of soil degradation, such as land desertification, salinization, soil erosion, or a given soil nutrient, there are few studies on comprehensive soil degradation. In addition, the data sources adopted by previous studies focused primarily on Landsat or other multi-spectral images, and near-ground hyperspectral data were used less frequently, so this study of hyper-spectral response and estimation of soil degradation has a certain research value and significance. Moreover, the hyperspectral data used in our study are from soil samples that were air-dried naturally indoors, and the effect is poor with spectral data of field soil samples, which requires further study.

## Conclusion

The SDI values of soil samples in the study area ranged from 0.2366 to 0.6590. Among these samples, 39% showed light degradation, 50% showed moderate degradation, and the remaining 11% showed heavy degradation. Salinization was the main factor in the soil degradation in Kenli County, and the vegetation cover types of the soil degradation grades from heavy to light were as follows: seepweed, reed, cogongrass, rice and cotton, and wheat, respectively.

The soil spectra of the different degradation grades were similar in shape, and as the grade of soil degradation increased, the overall quality of the reflectance spectra increased. The spectral information of soil degradation was more prominent in wavebands of larger spectral reflectance. The sensitive wavebands of soil degradation were found to be mainly near-infrared wavebands, and the first derivative transformation and waveband reorganization were able to enhance soil information and improve the correlation with SDI. The correlation between (R_λ2_+R_λ1_)/(R_λ2_-R_λ1_) and SDI was highest in all the types of spectral parameters, and the estimation model of SDI constructed by (R_λ2_+R_λ1_)/(R_λ2_-R_λ1_) was optimum. The optimum estimation model of SDI in this study was as follows:

$$\text{Y}=0.684-0.306\left({\text{R}}_{581}+{\text{R}}_{356}\right)/\left({\text{R}}_{581}-{\text{R}}_{356}\right)+0.065\left({\text{R}}_{934}+{\text{R}}_{599}\right)/\left({\text{R}}_{934}-{\text{R}}_{599}\right)+7.383\times 10^{-6}\left({\text{R}}_{2295}+{\text{R}}_{1726}\right)/\left({\text{R}}_{2295}-{\text{R}}_{1726}\right)-5.627\times 10^{-5}\left({\text{R}}_{2403}+{\text{R}}_{2011}\right)/\left({\text{R}}_{2403}-{\text{R}}_{2011}\right)-5.661\times 10^{-5}\left({\text{R}}_{2352}+{\text{R}}_{1459}\right)/\left({\text{R}}_{2352}-{\text{R}}_{1459}\right)-3.905\times 10^{-6}\left({\text{R}}_{2290}+{\text{R}}_{1996}\right)/\left({\text{R}}_{2290}-{\text{R}}_{1996}\right)+2.561\times 10^{-5}\left({\text{R}}_{2403}+{\text{R}}_{1721}\right)/\left({\text{R}}_{2403}-{\text{R}}_{1721}\right).$$

In this study, the spectral characteristics of coastal soil degradation were explored, and an estimation model of SDI was constructed. These results provide a positive reference for the utilization and management of land resources in the Yellow River Delta.
